# Supplementary material for: High-risk human papillomavirus genotypes among women of hill districts in Bangladesh
Source: PLoS One. 2025 Dec 8;20(12):e0338092. doi: 10.1371/journal.pone.0338092 (PMC12685158; doi:10.1371/journal.pone.0338092)
Supplement: S1 Text — (PDF) [file pone.0338092.s001.pdf]

Supplementary File S2.

PCR Primer Targets and Reagent Kits Used in the cobas® HPV Test

The **cobas® HPV Test** utilizes primers targeting an approximately 200-nucleotide sequence within the polymorphic L1 region of the HPV genome. A pooled set of primers amplifies DNA from 14 high-risk HPV genotypes (**16, 18, 31, 33, 35, 39, 45, 51, 52, 56, 58, 59, 66, and 68**). Fluorescent oligonucleotide probes hybridize to polymorphic regions within the amplified sequence to enable detection. An additional primer pair and probe target the **human β-globin gene** (330 bp amplicon) and serve as an internal process control to ensure sample adequacy and PCR efficiency.

A. Primer Targets

Target Region	Function	Product Size	HPV Types Detected	Notes
L1 region of HPV genome	Amplification of polymorphic sequence	~200 bp	14 high-risk types: 16, 18, 31, 33, 35, 39, 45, 51, 52, 56, 58, 59, 66, 68	Detected using fluorescent oligonucleotide probes
β-globin gene	Internal process control	330 bp	Human β-globin	Confirms sample adequacy and PCR efficiency

B. Reagent Kits Used in cobas® 4800 System

Kit Name	Description	Capacity	Product Number (P/N)
c4800 SMPL PREP	Sample Preparation Kit	960 Tests	05235804190
c4800 WB	Wash Buffer Kit	960 Tests	05235871190
c4800 LIQ CYT	Liquid Cytology Preparation Kit	960 Tests	05235839190
c4800 HPV AMP/DET	HPV Amplification/Detection Kit	960 Tests	05235898190
c4800 HPV CTLs	HPV Controls Kit	10 Sets	05235855190
