# Supplementary material for: High-risk human papillomavirus genotypes among women of hill districts in Bangladesh
Source: PLoS One. 2025 Dec 8;20(12):e0338092. doi: 10.1371/journal.pone.0338092 (PMC12685158; doi:10.1371/journal.pone.0338092)
Supplement: Attachment — Submitted filename: Response to reviewer.docx [file pone.0338092.s003.docx]

| **Comment Source** | **Comment / Requirement** | **Author Response / Action Taken** |
| --- | --- | --- |
| Journal Requirements – Style | Please ensure that your manuscript meets PLOS ONE's style requirements, including those for file naming. The PLOS ONE style templates can be found at <https://journals.plos.org/plosone/s/file?id=wjVg/PLOSOne_formatting_sample_main_body.pdf> and <https://journals.plos.org/plosone/s/file?id=ba62/PLOSOne_formatting_sample_title_authors_affiliations.pdf>. | Manuscript reformatted according to PLOS ONE style templates for main text and title/authors. File names updated per guidelines. |
| Journal Requirements – Overlapping text | We noticed you have some minor occurrence of overlapping text with the following previous publication(s), which needs to be addressed: Chakraborty S, Nessa A, Ferdous NE, Rahman MM, Rashid MHU, Sonia AA, Islam MF. Prevalence and genotypic distribution of high-risk human papillomavirus (HPV) among ever-married women in coastal regions of Bangladesh. PLoS One. 2024 Dec 12;19(12):e0313396. doi: 10.1371/journal.pone.0313396. PMID: 39666707; PMCID: PMC11637305. In your revision ensure you cite all your sources (including your own works), and quote or rephrase any duplicated text outside the methods section. Further consideration is dependent on these concerns being addressed. | All overlapping text rephrased; prior work properly cited; methods section text unchanged as allowed. |
| Journal Requirements – Financial disclosure | Thank you for stating the following financial disclosure: [The study was financially supported by the MOHFW of Bangladesh through the “Electronic Data Tracking with Population-based Cervical and Breast Cancer Screening Programme” (Code no. 16201-224259800). All the research activities, including were supported by he MOHFW’s funding covered cost of participant enrolment, data and HPV sample collection, transport cost, essential laboratory expenses, etc ensuring the successful implementation of the study.]. Please state what role the funders took in the study. If the funders had no role, please state: "The funders had no role in study design, data collection and analysis, decision to publish, or preparation of the manuscript." If this statement is not correct you must amend it as needed. Please include this amended Role of Funder statement in your cover letter; we will change the online submission form on your behalf. | The revised finding statement will be: " The study was financially supported by the MOHFW of Bangladesh through the  “Electronic Data Tracking with Population-based Cervical and Breast Cancer  Screening Programme” (Code no. 16201-224259800). All the research activities,  including were supported by he MOHFW’s funding covered cost of participant  enrolment, data and HPV sample collection, transport cost, essential laboratory  expenses, etc ensuring the successful implementation of the study. The funders had no role in study design, data collection and analysis, decision to publish, or preparation of the manuscript." |
| Journal Requirements – Expand MOHFW | Please expand the acronym “MOHFW” (as indicated in your financial disclosure) so that it states the name of your funders in full. This information should be included in your cover letter; we will change the online submission form on your behalf. | Expanded to full name: "Ministry of Health and Family Welfare, Bangladesh". |
| Journal Requirements – Acknowledgments | Thank you for stating the following in the Acknowledgments Section of your manuscript: [We pay our respect to the technical and financial support from of the Ministry of Health and Family Welfare (MOHFW) of Bangladesh, Special thanks are given to the health managers and healthcare providers at healthcare facilities at the district, sub-district, community clinics of the hill districts for their full cooperation. The authors are thankful to the colleagues of relevant departments of BMU. The authors would also like to express their gratitude to the participants of this study.] We note that you have provided funding information that is not currently declared in your Funding Statement. However, funding information should not appear in the Acknowledgments section or other areas of your manuscript. We will only publish funding information present in the Funding Statement section of the online submission form. Please remove any funding-related text from the manuscript and let us know how you would like to update your Funding Statement. | Funding-related text removed from acknowledgments; acknowledgments now focus on technical/logistical support and participant cooperation. |
| Journal Requirements – Data availability | In the online submission form, you indicated that [Most of the data collected were analyzed and are contained within this published article. To maintain data privacy, the data used are not publicly available. The data can be made available after a rational request from the researchers.]. All PLOS journals now require all data underlying the findings described in their manuscript to be freely available to other researchers, either 1. In a public repository, 2. Within the manuscript itself, or 3. Uploaded as supplementary information. This policy applies to all data except where public deposition would breach compliance with the protocol approved by your research ethics board. If your data cannot be made publicly available for ethical or legal reasons (e.g., public availability would compromise patient privacy), please explain your reasons on resubmission and your exemption request will be escalated for approval. | Updated Data Availability Statement: "Most of the data collected were analyzed and are included within this published article. To ensure participant privacy, the dataset is not publicly available but will be stored by the National Center for Cervical & Breast Cancer Screening & Training (NCCBCST). Data may be made available upon reasonable request to the corresponding authority via **viacbe@bsmmu.edu.bd**." |
| Journal Requirements – Cite prior work | If the reviewer comments include a recommendation to cite specific previously published works, please review and evaluate these publications to determine whether they are relevant and should be cited. There is no requirement to cite these works unless the editor has indicated otherwise. | All relevant prior studies reviewed and cited appropriately. |
| Reviewer #1 – Abstract language | The phrase “These findings direct policy makers” is awkward and should be rephrased. | Revised to: " These findings may guide policymakers to initiate HR-HPV DNA-based screening and reconsider vaccination strategies in the hill areas, including the introduction of gender-neutral vaccination." |
| Reviewer #1 – Partial genotyping | The term “partial genotyping” should either be briefly explained in the abstract or removed. | Revised as “Partial genotyping of HPV DNA specimens which detects the presence of 14 high-risk genotypes (including HPV-16, HPV-18, and others wasperformed at Bangladesh Medical University (BMU)” |
| Reviewer #1 – HPV16/18 | The authors should clearly specify that only HPV16 and HPV18 were genotyped individually, while the remaining types were detected as a pooled group. | Clarified in abstract and methods. |
| Reviewer #1 – Research gap | In the introduction, the research gap is not well justified. | Expanded introduction with references |
| Reviewer #1 – Sample size calculation | In the methods section, the sample size calculation is unclear. The assumed HPV prevalence of 7.1% appears arbitrary. | Assumed was removed and proper source cited |
| Reviewer #1 – Statistical analysis | The statistical analysis section contains repetitive descriptions and should be made more concise and precise. | Redundant text removed; section made concise. |
| Reviewer #1 – Tribal/non-tribal representation | The authors should clarify whether tribal and non-tribal women were proportionally represented across all three districts and explain how tribal identity was determined; was it self-reported? | Tribal identity was self-reported. |
| Reviewer #1 – Figures clarity / small HPV18 cases | Figures 3 and 4 lack clarity—axis labels and legends need improvement. Low number of HPV18-positive cases weakens subgroup analysis. | Figures revised; limitation regarding small HPV18-positive cases added. |
| Reviewer #1 – Logistic regression | Statistical concerns exist regarding the logistic regression analysis. With only 44 HR-HPV-positive cases, the model may be underpowered. | Statistical analysis section rewritten added information on model diagnostics and goodness-of-fit tests. Limitation acknowledged;. |
| Reviewer #1 – Cost-effectiveness claims | Assertions such as “screening and treatment will be cost-effective” need to be supported by data or citations. | Statements rephrased or citations added. |
| Reviewer #1 – Language/typos | The manuscript contains several grammatical and typographical errors, e.g., “leave in hard-to-reach areas” instead of “live.” | Manuscript edited thoroughly for English grammar and clarity. |
| Reviewer #1 – Data availability | Data availability statement is insufficient. PLOS ONE requires open and accessible data. | Revised as per journal requirements (see above). |
| Reviewer #2 – Informed consent | Nothing has been mentioned regarding taking a consent from the cohort of 1602 selected apparently healthy married women. | Written consent obtained; mentioned on the methods section |
| Reviewer #2 – Excluded participants | It would also be great to know what percentage of the people were excluded due to cervical precancer or known HPV-positive status. | Added: 11 women excluded. |
| Reviewer #2 – PCR primer details | Could you please provide all the scientific details regarding the PCR primers used during the experiment in the supplementary. | Complete added as Supplementary File S1 Text |
| Reviewer #2 – HPV types table | It would also be nice to create a small table regarding the different types of HPV like HPV16, , 18 and others. | Table 2 added summarizing HPV16, HPV18, and pooled other high-risk types. |
| Reviewer #2 – Conclusion | The conclusion can be made a bit stronger with these results. | Conclusion revised to emphasize prevalence, public health implications, and recommendations. |
